# Perspectives on Organelle Interaction, Protein Dysregulation, and Cancer Disease

**DOI:** 10.3389/fcell.2021.613336

**Published:** 2021-02-25

**Authors:** Paula Díaz, Alejandra Sandoval-Bórquez, Roberto Bravo-Sagua, Andrew F. G. Quest, Sergio Lavandero

**Affiliations:** ^1^Advanced Center for Chronic Diseases (ACCDiS), Faculty of Chemical and Pharmaceutical Sciences and Faculty of Medicine, Universidad de Chile, Santiago, Chile; ^2^Center for Studies on Exercise, Metabolism and Cancer (CEMC), Program of Cell and Molecular Biology, Faculty of Medicine, Institute of Biomedical Sciences (ICBM), Universidad de Chile, Santiago, Chile; ^3^Institute of Nutrition and Food Technology (INTA), Universidad de Chile, Santiago, Chile; ^4^Corporación Centro de Estudios Científicos de las Enfermedades Crónicas (CECEC), Santiago, Chile; ^5^Division of Cardiology, Department of Internal Medicine, University of Texas Southwestern Medical Center, Dallas, TX, United States

**Keywords:** interorganelle communication, cancer, mitochondria, endoplasmic reticulum, lysosome, peroxisome

## Abstract

In recent decades, compelling evidence has emerged showing that organelles are not static structures but rather form a highly dynamic cellular network and exchange information through membrane contact sites. Although high-throughput techniques facilitate identification of novel contact sites (e.g., organelle-organelle and organelle-vesicle interactions), little is known about their impact on cellular physiology. Moreover, even less is known about how the dysregulation of these structures impacts on cellular function and therefore, disease. Particularly, cancer cells display altered signaling pathways involving several cell organelles; however, the relevance of interorganelle communication in oncogenesis and/or cancer progression remains largely unknown. This review will focus on organelle contacts relevant to cancer pathogenesis. We will highlight specific proteins and protein families residing in these organelle-interfaces that are known to be involved in cancer-related processes. First, we will review the relevance of endoplasmic reticulum (ER)-mitochondria interactions. This section will focus on mitochondria-associated membranes (MAMs) and particularly the tethering proteins at the ER-mitochondria interphase, as well as their role in cancer disease progression. Subsequently, the role of Ca^2+^ at the ER-mitochondria interphase in cancer disease progression will be discussed. Members of the Bcl-2 protein family, key regulators of cell death, also modulate Ca^2+^ transport pathways at the ER-mitochondria interphase. Furthermore, we will review the role of ER-mitochondria communication in the regulation of proteostasis, focusing on the ER stress sensor PERK (PRKR-like ER kinase), which exerts dual roles in cancer. Second, we will review the relevance of ER and mitochondria interactions with other organelles. This section will focus on peroxisome and lysosome organelle interactions and their impact on cancer disease progression. In this context, the peroxisome biogenesis factor (PEX) gene family has been linked to cancer. Moreover, the autophagy-lysosome system is emerging as a driving force in the progression of numerous human cancers. Thus, we will summarize our current understanding of the role of each of these organelles and their communication, highlighting how alterations in organelle interfaces participate in cancer development and progression. A better understanding of specific organelle communication sites and their relevant proteins may help to identify potential pharmacological targets for novel therapies in cancer control.

## Introduction

Higher organisms are characterized by the cooperation between cell populations, which carry out different, complementary functions. This entails the existence of various gene expression programs encoded in the same genome, and processes such as cell differentiation and proliferation. All these processes must be dynamic and tightly regulated to maintain homeostasis in response to the ever-changing external and internal environments. However, multicellularity comes at a price: dysregulation of the aforementioned processes can lead to tumorigenesis and cancer (Trigos et al., 2018).

Cancer cells not only proliferate uncontrollably, but also they avoid differentiation and attain several special traits, such as the ability to increase the supply of nutrients, become invisible to the immune system, change their metabolism and adapt to surroundings that vary as the tumor progresses, among others (Fouad and Aanei, 2017). This transformation requires extensive genetic remodeling, which radically changes the intra and intercellular landscape.

Within the eukaryotic cell, organelles compartmentalize specific processes, which determines the cell phenotype and thus, have an impact on tumorigenesis. Relevant for this review, mitochondria play a key role in regulating cell death, as well as providing energy, which is, in turn, a major player in cell adaption and survival (Bravo-Sagua et al., 2017). The endoplasmic reticulum (ER), on the other hand, synthesizes large amounts of proteins and governs intracellular Ca^2+^ signaling, two activities crucial for cell viability (Oakes and Papa, 2015). Lysosomes, in turn, possess lytic enzymes required for the degradation of damaged organelles and other intracellular structures, serving as a quality control mechanism (Lawrence and Zoncu, 2019). This function involves a group of processes collectively termed autophagy (Lawrence and Zoncu, 2019). Finally, peroxisomes represent a set of vesicles that participate in lipid and oxidative metabolism, and are emerging players in cancer development (Islinger et al., 2018).

Since organelles harbor distinct and fundamental activities, they are meticulously modulated to preserve tissue physiology and avoid perturbations in homeostasis. Furthermore, organelles communicate with each other to coordinate stress responses, which entails damage-sensing at the ER surface by ER stress sensors, and increased ATP generation by mitochondria, fueled by metabolites generated through lysosome- and peroxisome-mediated degradation processes. One means of communication between them are interorganelle contacts, which permit direct exchange of signaling molecules and scaffolding of key regulatory complexes, thereby avoiding the fusion of their membranes and luminal continuity as a result (Lopez-Crisosto et al., 2015). Such is the importance of these contacts that their alteration is associated with various pathologies, especially cardiometabolic diseases (Lopez-Crisosto et al., 2017). ER-mitochondria contacts are the most widely studied organelle contacts, and, interestingly decline in most pathologies. This is consistent with the notion that lacking a coordinated response leads to maladaptation and, ultimately, cell dysfunction (Lopez-Crisosto et al., 2015).

Processes associated with cancer development and progression correlate with alterations in organelle communication (Boroughs and Deberardinis, 2015), although the nature of such dysregulation is far more complex and, in some cases, contradictory. This ambiguity in cancer may arise because its cause is not the lack of cell adaptation, but rather the opposite, namely the exacerbated capacity to overcome adversities at any cost (Fouad and Aanei, 2017). Thus, it follows that cancer cells have altered organelle interfaces, especially those regulating cell death and survival, adjustment to stress and removal, or even preservation of dysfunctional structures. However, while an intriguing concept, the evidence available supporting this notion is still rather scarce. Here, we provide a revision of the current literature, with the objective of revealing the relevance of organelle (mis)communication in the genesis of cancer disease.

## ER-Mitochondria Interactions

Mitochondria are double-membrane bound organelles, responsible for oxidative cell metabolism and reactive oxygen species (ROS) generation, Ca^2+^ homeostasis and apoptosis, among others (Wallace, 2012). Besides their own regulation, mitochondria interact and communicate with other organelles (Nunnari and Suomalainen, 2012). However, dysregulation in such communication may trigger aberrant mitochondrial function resulting in impaired energy metabolism and ion buffering. As a result, mitochondrial dysfunction plays an important role in cancer (Doghman-Bouguerra and Lalli, 2019).

Mitochondria interact with the ER mainly through membrane structures referred to as mitochondria-associated ER membranes (MAMs). MAMs are well-characterized, 10–30 nm organelle contact sites (Bernhard and Rouiller, 1956; Wieckowski et al., 2009) that are rich in Ca^2+^ transporters, enzymes participating in lipid synthesis and transport, as well as tumor suppressors and proteins encoded by oncogenes that regulate cell signaling pathways (Lee and Min, 2018). Thus, MAM-associated protein dysfunction is involved in tumorigenesis and tumor progression.

### Tethering Proteins and Proteins at the ER-Mitochondria Interphase and Their Role in Cancer Disease Progression

**FIGURE 1:**
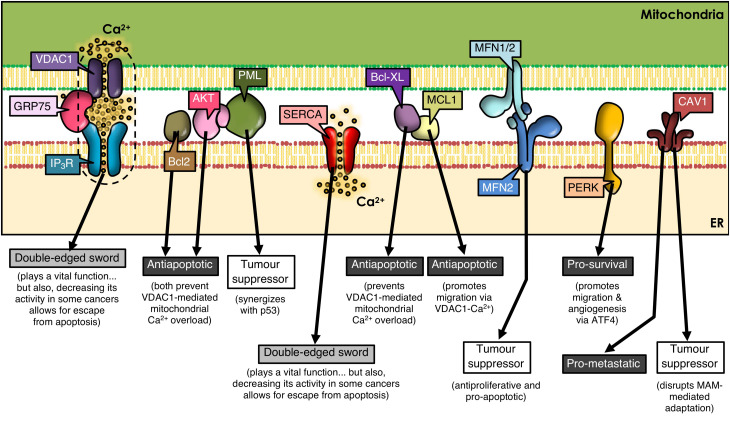
ER-mitochondria contacts and cancer development. Many proteins present at MAMs determine the outcome of tumorigenesis. VDAC1, GRP75, IP_3_R, and SERCA play a dual role, as they are essential for cell viability by mediating ER-to-mitochondria Ca^2+^ transfer; but, paradoxically, a decrease in their function prevents apoptosis in some cancer types. Many other MAM-residing proteins regulate IP_3_R-VDAC1 interplay, tilting the balance to one side or the other. Additional subsets of proteins are shown that either reside in or translocate to MAMs, thereby controlling signaling networks that modulate cell fate.

Mitofusins (MFN) are GTPases embedded in the ER surface and outer mitochondrial membrane (OMM), with a key role in mitochondrial dynamics. MFN regulates ER-mitochondria contacts between Ca^2+^-transfer sites (De Brito and Scorrano, 2008; Filadi et al., 2015; Naon et al., 2016). ER-resident MFN2 interacts with mitochondrial MFN1/MFN2 and regulates cell survival by playing anti-proliferative and pro-apoptotic roles (Ma et al., 2015; Wang et al., 2018b; Liu et al., 2019), as well as by participating in autophagy (Xue et al., 2018). MFN2 is considered a tumor suppressor (Figure 1) and is silenced in many malignant tumors (Zhang et al., 2013; Li et al., 2018). Thus, dysregulation of MFN2 function as a tethering protein at the ER-mitochondria interphase (De Brito and Scorrano, 2008, 2009) is likely to participate in cancer progression.

Caveolin-1 (CAV1), a membrane-associated scaffolding protein, that acts both as a tumor suppressor and a promoter of metastasis depending on the type of cancer and stage (Campos et al., 2019; Simon et al., 2020), is enriched in MAMs (Sala-Vila et al., 2016; Bravo-Sagua et al., 2019; Figure 1). There CAV1 plays a controversial role; on the one hand, CAV1 reportedly limits the adaptation to stress in tumor cells through impairment of ER-mitochondria contacts (Bravo-Sagua et al., 2019); on the other hand, using livers from wild-type and CAV1-deficient mice, it was shown that CAV1 promotes ER-mitochondria contacts, thereby contributing to the recruitment and regulation of intracellular steroids and lipoprotein metabolism (Sala-Vila et al., 2016). Whether these opposite effects may be related to the subcellular localization of CAV1 or the specific tumor cell-type, remains to be explored.

### The Role of Ca^2+^ at the ER-Mitochondria Interphase in Cancer Disease Progression

Ca^2+^ enters the mitochondria from the ER through MAMs where it regulates bioenergetics and metabolism by controling the activity of key enzymes of the tricarboxylic acid cycle and fatty acid (FA) oxidation (Glancy and Balaban, 2012). In addition, Ca^2+^ is important in mitochondrial fission and control of apoptosis. The low-affinity mitochondrial Ca^2+^ uniporter (MCU) receptor on the inner mitochondrial membrane (IMM) participates in Ca^2+^ uptake from the mitochondrial matrix and Ca^2+^ permeates to the OMM through the voltage-dependent anion channel 1 (VDAC1) (Baughman et al., 2011; Chaudhuri et al., 2013; Morciano et al., 2018). To do so, high local concentrations of Ca^2+^ need to be generated at MAMs, which are enriched in the Ca^2+^-sensitive inositol 1,4,5-trisphosphate receptor (IP_3_R). Ca^2+^ in the ER is rapidly released to the surrounding cytoplasm through IP_3_Rs, exposing mitochondria to higher concentrations of Ca^2+^ (Csordas et al., 2006, 2010). Alterations in the expression and/or function of these Ca^2+^-transport/binding systems have been implicated in oncogenesis and cancer progression (Marchi and Pinton, 2016; Bultynck and Campanella, 2017).

Several types of cancer cells undergo extensive reorganization of Ca^2+^ signaling to promote tumor progression. This reorganization of Ca^2+^-dependent mechanisms (i.e., Ca^2+^ transport pathways, Ca^2+^-dependent signaling) has a direct impact on cell survival by altering proliferation, migration, invasion, and metastasis (Chen et al., 2013; Monteith et al., 2017). Tumor cells display a dependency on constitutive Ca^2+^ transfer to maintain viability (Cardenas et al., 2016). For instance, the chaperone glucose regulatory protein 75 (GRP75), a protein expressed at MAMs, allows efficient IP_3_R-mediated Ca^2+^ transfer into mitochondria through VDAC on the OMM. However, upregulation of GRP75 in cancer cells has been associated with increased susceptibility to cell death (Wadhwa et al., 2006; Deocaris et al., 2007; Figure 1).

Factors that increase Ca^2+^ concentrations have been reported to upregulate VDAC expression and subsequently, the release of cytochrome C and Smac/Diablo, leading to apoptosis (Shoshan-Barmatz et al., 2017). Mcl-1, an anti-apoptotic member of the Bcl-2 family, binds with high affinity to VDAC1 and promotes lung cancer cell migration by a mechanism that involves Ca^2+^-dependent ROS production (Huang et al., 2014; Figure 1). Moreover, the BH4 domain of Bcl-XL, selectively targets VDAC1 and inhibits apoptosis by decreasing VDAC1-mediated Ca^2+^ influx into the mitochondria (Monaco et al., 2015; Figure 1). These reports underscore the relevance of highly regulated MAM proteins in ER-mitochondrial Ca^2+^ transfer, and implicate their dysregulation in tumorigenesis.

The activity of the sarco/ER Ca^2+^-ATPase (SERCA) pump is, among others, regulated by proteins encoded by oncogenes and tumor suppressor proteins in MAMs (Vandecaetsbeek et al., 2011). Particularly, the SERCA2b subtype is highly abundant in MAMs (Lynes et al., 2012; Chemaly et al., 2018) and the tumor suppressor p53 controls SERCA2b activation to promote Ca^2+^-dependent apoptosis (Mcdonnell et al., 2019). Thus, the ER releases increased amounts of Ca^2+^, which enters the mitochondria causing Ca^2+^ overload and apoptosis. However, in cancer cells, p53 at MAMs is mutated or inactivated, and thus, the ER cannot maintain elevated Ca^2+^ levels, which allows cancer cells to escape apoptosis contributing to tumor progression (Giorgi et al., 2015; Figure 1).

The complete inhibition of IP_3_R activity results in markedly compromised mitochondria bioenergetics and increased vulnerability to cell death and reduced melanoma tumor growth (Cardenas et al., 2016). Moreover, proteins encoded by oncogenes and tumor suppressors modulate ER IP_3_R activity in MAMs, thus altering Ca^2+^ signaling in cancer cells (Fan et al., 2017).

For instance, IP_3_R phosphorylation is markedly increased in cancer cells by hyperactive Akt (Szado et al., 2008), thereby reducing Ca^2+^ release from the ER to mitochondria, and promoting tumor survival (Szado et al., 2008). Other studies have shown that promyelocytic leukemia protein (PML), a tumor suppressor which localizes to MAMs (Giorgi et al., 2010), forms a complex with Akt, and decreases binding of protein phosphatase 2A (PP2A) to IP_3_Rs, suggesting that PP2A no longer dephosphorylates and inactivates Akt. This leads to phosphorylation of Akt and IP_3_Rs, decreasing Ca^2+^ release and further protecting the mitochondria from Ca^2+^-mediated apoptosis (Marchi et al., 2012). Moreover, PML physically interacts and synergizes with tumor suppressor protein p53 during apoptosis (Bernardi et al., 2008; Figure 1). Deletion of PML is associated with pleural mesothelioma and breast cancer, among others (Plevova et al., 2007a, b; Wang et al., 2018c). Additionally, Bcl-2 family members in the ER regulate IP_3_R activity (Lian et al., 2018) and, consequently apoptosis, by controlling the release of cytochrome C, the integrity of mitochondrial membranes and the activation of caspases (Youle and Strasser, 2008). Other evidence indicates that Bcl-2 binds to and inhibits IP_3_Rs, which reduces Ca^2+^ release, leading to decreased apoptosis (Rong et al., 2009). In summary, the dysregulation of proteins involved in Ca^2+^ transport at MAMs or in oncogenes and tumor suppressor proteins in cancer, alters Ca^2+^ transfer from the ER to the mitochondria, and, whether the outcome is the inhibition of Ca^2+^ transfer or Ca^2+^ overload, it will contribute directly to cancer disease progression by modulating cell death and/or survival.

### The Role of ER Stress at the ER-Mitochondria Interphase in Cancer Disease Progression

ER stress is emerging as an important modulator of different pathologies and as a mechanism contributing to cancer cell death (Oakes and Papa, 2015). PERK (PRKR-like ER kinase), a key ER stress sensor of the unfolded protein response (UPR), is uniquely enriched at MAMs (Verfaillie et al., 2012). As a large number of molecular chaperones assist in the folding of unfolded proteins during the UPR, they consume large amounts of ATP. In order to increase ATP generation, cells usually increase the contact area between ER and mitochondria, which in turn increases Ca^2+^ release from the ER to the mitochondria (Bravo et al., 2012). However, if ER stress becomes chronic, ER-mitochondrial contacts and ER Ca^2+^ release increases, which, together with mitochondrial Ca^2+^ influx, leads to apoptosis. Moreover in cancer cells, the UPR is constitutively activated. It has been reported that PERK plays a critical role in tumor invasion and metastasis (Jamison et al., 2015; Pytel et al., 2016; Rozpedek et al., 2016; Feng et al., 2017). PERK signaling, which is activated downstream of the UPR and the integrated stress response (ISR), is triggered in response to a range of pathophysiological changes, and enables cancer cells to survive the adverse conditions typically observed in the tumor microenvironment. The ISR can be induced by both, intrinsic (i.e., ER stress) and extrinsic factors. The latter include hypoxia, amino acid and glucose deprivation, among others (Pakos-Zebrucka et al., 2016). Within the ISR, different protein kinases phosphorylate the α-subunit of eukaryotic initiation factor-2 (eIF2α), including heme-regulated eIF2α kinase (HRI), general control non-depressible protein 2 (GCN2), double stranded RNA dependent protein kinase (PKR) and PERK (Pakos-Zebrucka et al., 2016; Wang et al., 2018a). Phosphorylation of eIF2α inhibits global protein translation and synthesis, thereby reducing the number of proteins entering the ER (Baird and Wek, 2012), but increasing the cap-independent translation of other mRNAs, such as activating transcription factor 4 (ATF4) (Vattem and Wek, 2004). In addition to enabling cell survival, PERK-ATF4 signaling triggers multiple steps in the metastatic cascade, including angiogenesis, migration, survival, and colonization of secondary organ sites (Feng et al., 2017). PERK is also required for the metastatic dissemination of cancer cells that have undergone epithelial-to-mesenchymal transition (Feng et al., 2017; Figure 1). Thus, while PERK contributes to tighter MAMs during ER stress-induced apoptosis (Verfaillie et al., 2012), other roles of MAM resident-PERK and their contribution to cancer disease progression remain unclear.

## ER and Mitochondria Interactions With Other Organelles

Peroxisomes are metabolic organelles that carry out vital cell functions in lipid metabolism and synthesis, as well as maintenance of the redox balance (Islinger et al., 2018). Furthermore, non-metabolic roles of peroxisomes have been discovered, relating to cellular stress responses and the regulation of immune responses (Dahabieh et al., 2018). Disturbances between membrane contact sites (MCSs), areas of close proximity between the membranes of two organelles, can cause diseases (Scorrano et al., 2019). Most of the pathways that involve peroxisomes require communication with other organelles, such as the ER, mitochondria, lipid droplets and lysosomes, whereby close proximity represents a prerequisite for the efficient transport of metabolites (Sargsyan and Thoms, 2020). The main metabolic pathways shared between peroxisomes and the ER are: peroxisomal β-oxidation, implicated in the degradation of FA and biosynthesis of docosahexaenoic acid and the bile acids, ether lipid synthesis; and membrane lipid transfer (Ferdinandusse et al., 2018). Those shared between peroxisomes and mitochondria are: glyoxylate detoxification, redox exchange, ROS metabolism (Meo-Evoli et al., 2015) and α-oxidation (Michiels et al., 2016). Other MCSs exist that are implicated in the transfer of hydrophobic molecules, such as FA, between lipid droplets and peroxisomes (Valm et al., 2017), as well as cholesterol from or to lysosomes (Chu et al., 2015). The interaction of peroxisomes with the surrounding organelles begins with their biogenesis. Peroxisome formation involves generating a lipid membrane, followed by the acquisition of peroxisome membrane proteins, and subsequently organelle expansion (Farre et al., 2019). The peroxisomes originate from ER-derived vesicles containing the biogenesis factors (PEX) PEX3 and PEX16, or by fusion of PEX-bound vesicles from both, mitochondria (PEX3 and PEX14 factors), and the ER (Sugiura et al., 2017).

**FIGURE 2:**
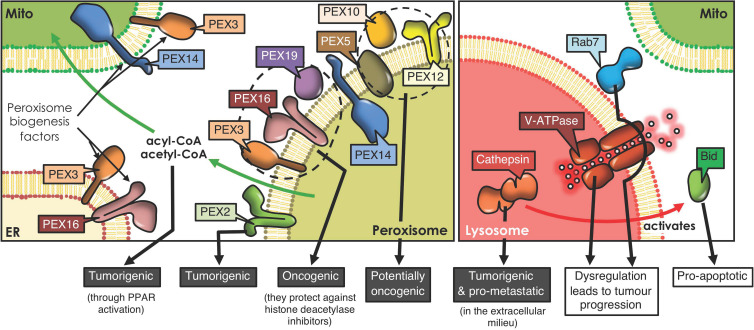
Peroxisome and lysosome interactions and cancer development. Peroxisomes derive from ER and mitochondrial membranes, and the proteins involved in peroxisome biogenesis and their regulation reportedly have tumorigenic potential. Moreover, peroxisome-mitochondria metabolic coupling favors cell proliferation and tumorigenesis through fatty acid transfer. Lysosomes form contacts with mitochondria, orchestrated by proteins, such as Rab7. Lysosomes play an ambiguous role in cancer. For instance, cathepsins, the proteolytic lysosomal enzymes, promote tumorigenesis when released to the extracellular milieu, but trigger Bid-mediated apoptosis upon accessing the cytoplasm. Also, dysregulation of either V-ATPase, the complex that acidifies lysosomes, or Rab7, the GTPase that orchestrates mitochondria-lysosome contact sites, both promote tumor progression (PPAR, Peroxisome proliferator-activated receptor).

In recent years, considerable information has emerged suggesting that dysregulation of peroxisomes is implicated in tumorigenesis (Dahabieh et al., 2018). Enzymes implicated in peroxisomal metabolic processing are dysregulated in numerous neoplasms, including gastric adenocarcinoma (Jindal et al., 2016), cervical cancer (Wu et al., 2018), prostate cancer (Valenca et al., 2015), ovarian cancer (Sun et al., 2018), colorectal neoplasia (Shukla et al., 2017), liver cancer (Chen et al., 2018) and glioblastomas (Ruokun et al., 2016). A recent study reports that silencing PEX2, a peroxin involved in autophagosomal degradation of peroxisomes (pexophagy), reduced tumor growth in liver cancer (Cai et al., 2018). These results suggest that PEX2 and other peroxins, such as PEX5, 10 and 12, are essential for the viability of tumor cells and, thus, represent potential therapeutic targets for the treatment of cancer (Islinger et al., 2018; Figure 2). Another study revealed that PEX3, PEX16, and PEX19 protect lymphoma cells against histone deacetylase inhibitor induced-cell death, thereby promoting tumorigenesis (Dahabieh et al., 2017; Figure 2). These findings highlight a possible contribution of peroxisomes in chemotherapy resistance. Moreover, peroxisomes participate in the secretion of immune system modulators (e.g., interleukin-1, tumor necrosis factor) and lipids with pro- and anti-inflammatory characteristics, all of which are connected to tumor-promoting inflammation (Di Cara et al., 2019).

Another relevant role of peroxisomes in malignancy is attributable to their crosstalk with mitochondria. Following peroxisomal β-oxidation, the final products, acyl-CoA and acetyl CoA, can enter the mitochondrial pathway and activate peroxisome proliferator-activated receptors, which are implicated in tumorigenesis (Dahabieh et al., 2018; Figure 2). In addition, dysfunction of peroxisomes leads to other metabolic alterations, through deficiency of peroxins (Faust and Kovacs, 2014). PEX2-depleted cells show elevated oxidative stress and elevated ROS causing inhibition of mechanistic target of rapamycin complex 1 (mTORC1) signaling, thereby promoting autophagy (Cai et al., 2018). This mechanism limits cancer initiation in some neoplasms; but in others, cancer cells use recycled metabolites to preserve organelle function and energy homeostasis to meet increased metabolic requirements. Thus, in these cells, autophagy is an essential survival mechanism to maintain cellular growth and proliferation (White et al., 2015).

Lysosomes, together with the ER and Golgi apparatus, are members of a network of intracellular membranous organelles whose functions are essential to maintain cell homeostasis (Buratta et al., 2020). Lysosomes degrade and recycle macromolecules via endocytosis, phagocytosis, and autophagy (Piao and Amaravadi, 2016; Lawrence and Zoncu, 2019). Moreover, they participate in extracellular events by secreting their contents through fusion with the plasma membrane (Davidson and Vander Heiden, 2017). Thus, lysosomes are extremely dynamic organelles, essential in a variety of cellular processes, such as cell signaling, death, immunity, and stress responses (Piao and Amaravadi, 2016). The dysregulation of these functions plays an important role in tumorigenesis, suggesting that lysosomal function is critical in this context. In fact, numerous hallmarks of cancer may be acquired as a result of lysosomal dysfunction (Hanahan and Weinberg, 2011). Specifically, the release of lysosomal proteases may activate caspases and lead to cell death; however, inhibition of lysosomal function, which hinders clearance of dead cells, contributes to inflammation and promotes tumorigenesis (Davidson and Vander Heiden, 2017). In addition, changes in the interactions between lysosomes and other organelles can limit organellophagy, thereby affecting the recycling of macromolecules and consequently, altering cellular energetics (Boroughs and Deberardinis, 2015). These changes include, the dysregulation of specific receptors, such as members of the FAM134 reticulon protein family in the ER (Hanahan and Weinberg, 2011; Khaminets et al., 2015). Overall, numerous hallmarks of cancer may be acquired as a result of lysosomal dysfunction and changes in the communication between lysosomes and other organelles (Hanahan and Weinberg, 2011).

Lysosomal and mitochondrial functions are connected (Plotegher and Duchen, 2017), since damaged mitochondria are engulfed by autophagosomes (Lazarou et al., 2015), which then fuse with the lysosome/late endosome to produce autolysosomes where the mitochondria are degraded (mitophagy) (Wong et al., 2019). In addition, mitochondria-derived vesicles may fuse with lysosomes to eliminate their contents (Plotegher and Duchen, 2017). In doing so, toxic accumulation of damaged proteins, especially in the mitochondria, which augment oxidative stress and activate oncogenic signaling, can be avoided (White, 2015). On the other hand, lysosome-mitochondria interactions may also contribute to cancer initiation (Ferguson, 2015). Cell transformation results in an elevated demand for nutrients, associated with the increased production of cell mass, and lysosomes may provide the required molecules together with autophagy, to preserve mitochondrial functions and energy homeostasis (White et al., 2015). Additionally, lysosomes and mitochondria are connected via non-degradative processes involving the formation of dynamic interorganelle MCSs (Wong et al., 2019). These processes are regulated by Rab7, which controls this organelle network, as well as processes including mitochondrial fission (Yamano et al., 2018; Figure 2). Changes in interorganelle transfer of metabolites may alter cell homeostasis and, consequently, favor the development of diseases linked to dysfunction of both organelles, such as cancer (Wong et al., 2019). For instance, it has been reported that cytosolic cathepsins (lysosomal hydrolases) may repress tumor growth by activation of an intrinsic apoptotic pathway. Contrarily, extracellular cathepsins (e.g., B, S, and E) may stimulate tumor growth, progression and metastasis in different neoplasms by aiding in permeating the basement membrane and activating pro-tumorigenic proteins (Piao and Amaravadi, 2016; Figure 2). Additionally, the V-ATPase (lysosomal membrane protein), a proton pump that modulates intravesicular acidification in lysosomes, is an important regulator of endocytic trafficking (Plotegher and Duchen, 2017). A recent study reveals that V-ATPase is a master effector of transcription factor E2F1-mediated lysosomal trafficking, essential in mTORC1 activation and suppression of autophagy, which promotes tumor progression (Meo-Evoli et al., 2015; Figure 2).

Furthermore, lysosomal membrane permeabilization (LMP; loss of membrane integrity), causes the release of lysosomal enzymes into the cytosol, triggering apoptosis, autophagy and necroptosis (Piao and Amaravadi, 2016). LMP can accelerate apoptosis through cathepsin-mediated cleavage of Bid, to promote the release of cytochrome C via Bax (intrinsic pathway activation) (Wang, 2015; Piao and Amaravadi, 2016; Figure 2).

A number of cellular processes in which lysosomes participate have been identified as potential therapeutic targets in cancer. These include drugs that permeabilize lysosomal membranes, cathepsin inhibitors, and molecules that become protonated in order to increase the concentration of specific drugs inside the organelle. However, until now, related clinical data is scarce (Davidson and Vander Heiden, 2017). Thus, future studies on lysosomes and their interaction with other organelles will be critical to understanding the role that lysosome-communication plays in cancer genesis and progression.

## Conclusion

Organelles are the major players in intracellular communication networks. Multiple diseases have been studied by analyzing organelles individually, as is the case for mitochondria or the ER. Advances in microscopy have provided further insights to the nature of organelle communication; revealing both, physical and functional interactions at MCSs (Csordas et al., 2010; Valm et al., 2017; Shai et al., 2018).

Currently there is a consensus that organelles contact one another and communicate in a dynamic fashion. Over the last decade, evidence highlighting the importance of organelle communication in disease has become available (Bravo-Sagua et al., 2014; Lopez-Crisosto et al., 2015, 2017; Plotegher and Duchen, 2017; Torres et al., 2017). Moreover, organelle communication impacts on various cancer hallmarks and therefore plays a critical role in oncogenesis. This is the case of CAV1, a protein located at MAMs that acts both as a tumor suppressor and a promoter of metastasis (Campos et al., 2019; Simon et al., 2020). On the other hand, the ER stress sensor PERK, also present in MAMs, has been associated with apoptotic cell death and the promotion of more aggressive/migratory phenotypes. Additionally, the main peroxisome-organelle interactions so far studied involve peroxins, which also behave as tumorigenic factors. Lastly, cathepsins have been described in lysosomes as proteins that contribute to tumor progression and cell death. Overall, the details of how such communication between organelles contributes to cell homeostasis often still remain poorly understood.

The development of molecules that target organelle interactions at a sub-cellular level, represents a promising field in cancer treatment (Piao and Amaravadi, 2016; Kerkhofs et al., 2018). However, to exploit this potential a better understanding of the differences between interorganelle contacts in healthy and tumor cells is required.

## Author Contributions

PD, AS-B, and RB-S drafted the manuscript with support from AFGQ and SL. RB-S drafted the figures. PD, AS-B, RB-S, AFGQ, and SL critically revised the article. All authors read and approved the final article.

## Conflict of Interest

The authors declare that the research was conducted in the absence of any commercial or financial relationships that could be construed as a potential conflict of interest.
